# The UPTAKE study: implications for the future of COVID-19 vaccination trial recruitment in UK and beyond

**DOI:** 10.1186/s13063-021-05250-4

**Published:** 2021-04-20

**Authors:** Sonika Sethi, Aditi Kumar, Anandadeep Mandal, Mohammed Shaikh, Claire A. Hall, Jeremy M. W. Kirk, Paul Moss, Matthew J. Brookes, Supratik Basu

**Affiliations:** 1 The Royal Wolverhampton NHS Trust, New Cross Hospital, Wolverhampton Road, Wolverhampton, West Midlands WV10 0QP UK; 2 University of Birmingham, Birmingham, UK; 3 National Institute for Health Research, Clinical Research Network, Coventry, West Midlands UK; 4 Research Institute in Healthcare Sciences (RIHS), University of Wolverhampton, Wolverhampton, UK

**Keywords:** COVID-19, Vaccination trials, Population survey, BAME, Vaccine

## Abstract

**Background:**

Developing a safe and effective vaccine will be the principal way of controlling the COVID-19 pandemic. However, current COVID-19 vaccination trials are not adequately representing a diverse participant population in terms of age, ethnicity and comorbidities. Achieving the representative recruitment targets that are adequately powered to the study remains one of the greatest challenges in clinical trial management. To ensure accuracy and generalisability of the safety and efficacy conclusions generated by clinical trials, it is crucial to recruit patient cohorts as representative as possible of the future target population. Missing these targets can lead to reduced validity of the study results and can often slow down drug development leading to costly delays.

**Objective:**

This study explores the key factors related to perceptions and participation in vaccination trials.

**Methods:**

This study involved an anonymous cross-sectional online survey circulated across the UK. Statistical analysis was done in six phases. Multi-nominal logistic models examined demographic and geographic factors that may impact vaccine uptake.

**Results:**

The survey had 4884 participants of which 9.44% were Black Asian Minority Ethnic (BAME). Overall, 2020 (41.4%) respondents were interested in participating in vaccine trials; 27.6% of the respondents were not interested and 31.1% were unsure. The most interested groups were male (OR = 1.29), graduates (OR = 1.28), the 40–49 and 50–59 age groups (OR = 1.88 and OR = 1.46 respectively) and those with no health issues (OR = 1.06). The least interested groups were BAME (OR = 0.43), those from villages and small towns (OR = 0.66 and 0.54 respectively) and those aged 70 and above (OR = 1.11).

**Conclusions:**

In order to have a vaccination that is generalisable to the entire population, greater work needs to be done in engaging a diverse cohort of participants. Public health campaigns need to be targeted in improving trial recruitment rates for the elderly, BAME community and the less educated rural population.

## Highlights

- This study investigated demographic factors in COVID-19 vaccine trial participation.
- Our results show that only 41.4% are interested in partaking in vaccination trials.
- Male graduates and with no health issues are most interested in trial participation.
- Participation interest is lowest from rural and high-risk groups (BAME and elderly).
- Recruitment challenges will remain as phase 3 trials continue in 2021.

## Background

COVID-19 is an infectious respiratory condition that is caused by severe acute respiratory syndrome coronavirus 2 (SARS-CoV-2) [1]. It was initially detected in Wuhan in November 2019 and has rapidly become a global pandemic. As of 1st April 2021, it has infected 129 million people and caused 2.8 million deaths globally [2]. Evidence has established that COVID-19 is mainly spread via droplets and contact, with clinical characteristics of a febrile and inflammatory biphasic illness with associated respiratory tract inflammation [3, 4]. Of notable concern is how asymptomatic people are playing a major role in the transmission of this virus as it seems to shed at high concentrations from the nasal cavity before symptom development [5].

The first wave of the pandemic led to worldwide curfews and social distancing restrictions to prevent the further spread of COVID-19, with as many as 3.9 billion people being in some form of lockdown in the first week of April 2020. A second wave of lockdown measures was re-introduced for most of Europe and Asia in October and November 2020, placing a huge burden on the economy and people’s mental and physical wellbeing [6].

Ultimately, developing a vaccine will be the principal way of controlling this pandemic and curbing the COVID-19 death toll. As of yet, there are currently no licenced vaccines for COVID-19. Before a vaccine can be manufactured, distributed and administered to the population, it must first be deemed safe and effective [7]. Clinical trials are considered to be the most reliable and traditional way of testing a new vaccine [8]. Reaching the representative recruitment targets that are adequately powered to the study remains one of the greatest challenges in clinical trial management. Only approximately 50% of vaccine clinical trials in the UK achieve their recruitment target, resulting in approximately one third of trial terminations [9].

Thus, it is crucial to recruit a panel of patients as representative as possible of the future target population. This is to ensure the accuracy and generalisability of the efficacy and safety conclusions generated by clinical trials [10]. The vaccine trials should also represent a diverse participant population in terms of age, ethnicity and comorbidities. Missing these targets can lead to a reduced validity of the study results and can often slow down drug development leading to costly delays [11].

This study explores the key factors related to perceptions and participation in vaccination trials. Understanding the demographics of those who are less likely to partake in trials will help to target strategies in recruiting patients to trials.

## Methods

### Survey design and study population

This study was developed as a national anonymous cross-sectional online survey to help inform service decision-making. The survey was in English, hosted via Google forms and was open from 4th September 2020 to 9th October 2020. The survey was circulated across the UK through social media networks (Facebook, Twitter, LinkedIn and Instagram), national radio, news articles, the National Institute for Health Research (NIHR) Clinical Research Network West Midlands (CRN WM) website and newsletter and through 150 general practices via a text messaging service. Particular focus was put into targeting BAME groups through targeting BAME-specific social media and media outlets.

The interview questions were externally reviewed by the CRN WM Equality, Diversity and Inclusion Research Champions Group. Feedback from this group led to changes being made to the questions in order to make them more easily understandable, prior to the survey going live.

The survey consisted of several sections. First, Likert rating scales were used to determine the extent of agreement regarding various statements about COVID-19 and vaccinations. The survey then focused on previous vaccination habits (e.g. if the respondent had ever declined vaccinations) and whether they would be interested in taking part in the trial. The final section collected the respondent’s demographics. Once they had completed the survey, respondents were provided with a link to the NIHR ‘Be Part of Research’ website, as to how they could find out more about participating in the trials. The full survey can be found in Additional file 1.

This study was approved by local approval processes by the CRN WM. No ethical-related issues were identified. Participants were provided with information about the study and how the data was going to be disseminated in the initial page of the survey. This was an entirely anonymous survey with no identifiable material or information collected. Specific or individual consent was not obtained as the patients were participating without providing any identifiable material.

### Statistical analysis

The statistical analysis was done in six phases. The first described the data of the participants of the COVID-19 survey, including the various factors considered in the analysis. In the second phase, a postcode analysis was conducted to explore the regional variations in participation of the vaccine trials. Postcodes were classified into six subgroups (i.e. core city, other cities, large town, medium town, small town and village) based on the UK government’s postcode classification for population distribution. ‘Core cities’ include 12 major population and economic centres (London, Birmingham, Glasgow, Liverpool, Bristol, Manchester, Sheffield, Leeds, Edinburgh, Cardiff, Nottingham and Newcastle-upon-Tyne). The other subgroups are based on settlement population: ‘other cities’ greater than 175,000, ‘large town’ between 60,000 and 174,999, ‘medium town’ between 25,000 and 59,999, ‘small town’ between 7500 and 24,999, and ‘village’ below 7500 [12].

The third phase investigated the various factors influencing the respondents’ interest in vaccination trials. This analysis was done using a multinomial logistic regression model to estimate odds ratio for these factors. This included age, gender, ethnicity (BAME and non-BAME), diagnosed health condition, smoking status and qualification. The fourth phase examined those who were ‘not-interested’ in trial participation group, especially focusing on the impact of ethnicity and age. In the fifth phase, the differences in the mean scores of the questionnaire were explored to examine how it varied across participants who were interested in trials. Finally, in the last phase, a principal component analysis was used to explore the perception of vaccine and its effect on participation in vaccine trials. All analysis was carried out in STATA version 16.

## Results

A total of 4884 respondents completed the survey. The majority were females (*n* = 3416, 69.9%) and of White ethnicity (*n* = 4127, 84.5%). There were 461 BAME respondents (9.44%), amongst which 258 (5.3%) were Asian/Asian British-Indian and 67 (1.38%) were Black/African/Caribbean/Black British. The majority of the respondents were qualified up to at least A-level (*n* = 1574, 32.2%), with 1780 (36.4%) university undergraduate degree holders and 1010 (20.7%) post-graduate respondents. The age group between 50 and 59 years was the largest participant age group (1101 responses, 22.5%), with 552 (11.3%) responses from those aged 70 and above. 39.9% (*n* = 1949) of the respondents stated diagnosed health issues.

Overall, 2020 (41.4%) respondents were interested in participating in vaccine trials. 27.6% (1348) of the respondents were not interested in vaccine trials and 31.1% (1518) were unsure. See Table 1 for the full breakdown.

**Table 1:** Descriptive of survey participants

	Respondents	Percent (%)
**Interested in COVID-19 vaccine trials**		
Interested	2020	41.4
Not interested	1346	27.6
Unsure	1518	31.1
**Age group**		
Under 18	7	0.1
18–29	525	10.7
30–39	708	14.5
40–49	1042	21.3
50–59	1101	22.5
60–69	914	18.7
70 and above	552	11.3
Prefer not to say	35	0.7
**BAME community**		
Non-BAME	4374	89.6
BAME	461	9.4
Prefer not to say	49	1.0
**Qualification**		
No formal qualifications	127	2.6
Up to A-level	1574	32.2
University degree (under graduation)	1780	36.4
Post-graduation	1010	20.7
Prefer not to say	393	8.0
**Gender**		
Female	3416	69.9
Male	1426	29.2
Prefer not to say	42	0.9
**Ethnicity**		
White-English/Welsh/Scottish/Northern Irish/British	4127	84.5
White-Irish	49	1.0
White-Gypsy or Irish Traveller	3	0.1
White-Roma	2	0.0
White-others	193	4.0
Asian/Asian British-Indian	258	5.3
Asian/Asian British-Pakistani	30	0.6
Asian/Asian British-Chinese	19	0.4
Asian/Asian British-Bangladeshi	18	0.4
Mixed/multiple ethnic groups	69	1.4
Black/African/Caribbean/Black British-African	67	1.4
Prefer not to say	49	1.0
**Smoker**		
Smoker	386	7.9
Non-smoker	4495	92.0
Prefer not to say	3	0.1
**Diagnosed health issue**		
No-health issue	2935	60.1
Health issue	1949	39.9

The results presented in Table 2 shows that maximum participation was from ‘other cities’ (29.07%) followed by ‘small town’ (22%). Of those respondents not interested in participating in vaccine trials, the majority were from ‘villages’ (31.95%) whilst 46.18% (*n* = 701) of the respondents who were unsure were from ‘small towns’. Table 3 shows that whilst the maximum number of male respondents interested in trials were from ‘medium town’ (248, 35.6%), the majority of the interested females were from ‘other city’ (494, 37.5%). Whilst the majority of the graduates and post-graduates who were interested in trials were from ‘other city’, a significant number of non-university goers who were interested in trials were from ‘medium town’. Respondents aged 50 and less who were interested in vaccine trials lived in ‘other city” whilst those aged 60 and over that were interested in trials came from ‘medium town’.

**Table 2:** Postcode classification of respondents

Postcode classification	Interested (%)	Not interested (%)	Unsure (%)	Total (%)
**Core city**	304 (15.05)	252 (18.72)	0 (0.00)	556 (11.38)
**Other city**	**698 (34.55)**	144 (10.70)	578 (38.08)	**1420 (29.07)**
**Large town**	148 (7.33)	9 (0.67)	0 (0.00)	157 (3.21)
**Medium town**	592 (29.31)	153 (11.37)	34 (2.24)	779 (15.95)
**Small town**	17 (0.84)	358 (26.60)	**701 (46.18)**	1076 (22.03)
**Village**	261 (12.92)	**430 (31.95)**	205 (13.50)	896 (18.35)
**Total respondents**	2020 (41.36)	1346 (27.56)	1518 (31.08)	4884 (100)

**Table 3:** Interested in trials (postcode breakup)

Interested in trials	Postcode classifications						Total respondents	Percentage
	Core city	Other city	Large town	Medium town	Small town	Village		
**Gender (%)**								
Male	77	203	60	248	6	102	696	34.46
Female	223	494	88	343	11	159	1318	65.25
Prefer not to say	4	1	0	1	0	0	6	0.30
**Smoker (%)**	17	50	7	62	0	28	164	8.12
**Diagnosed health condition (%)**	102	300	58	243	9	119	831	41.14
**Qualification (%)**								
No qualification	4	14	3	24	0	9	54	2.67
Non-university goers	79	198	47	241	6	84	655	32.43
Graduates	118	262	53	197	7	102	739	36.58
Post-graduates	89	190	33	94	4	41	451	22.33
Prefer not to say	14	34	12	36	0	25	121	5.99
**Age group (%)**								
Under 18	0	2	0	1	0	1	4	0.20
18–29	28	80	11	30	3	25	177	8.76
30–39	72	89	16	60	2	23	262	12.97
40–49	97	161	35	92	2	49	436	21.58
50–59	57	163	44	151	4	67	486	24.06
60–69	32	137	31	153	4	65	422	20.89
70 +	18	65	11	104	2	31	231	11.44
Prefer not to say	0	1	0	1	0	0	2	0.10
**Ethnicity (%)**								
BAME	43	46	8	13	1	5	116	5.74
Non-BAME	258	650	140	578	16	256	1898	93.96
Prefer not to say	3	2	0	1	0	0	6	0.30

**Fig. 1:**
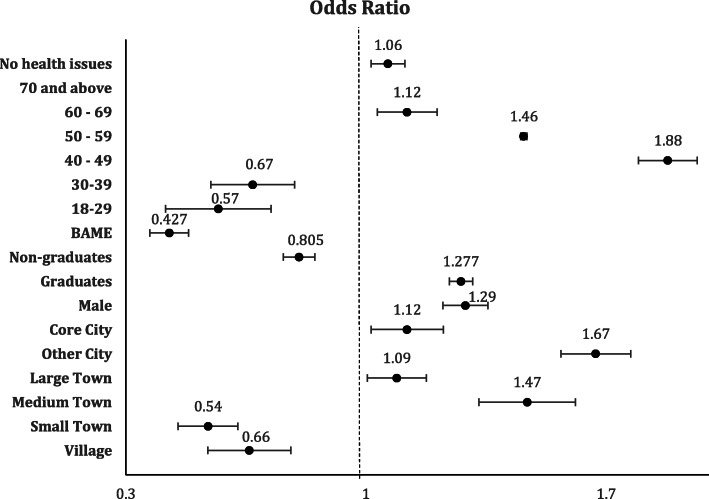
Factors influencing ‘Interest in Vaccine Trials’ (in colour)

Figure 1 presents the chart-view of the odds ratio (OR) of the various factors that significantly influence respondent’s interest in participating in COVID-19 vaccine trials. The results indicate that respondents from ‘village’ (OR = 0.66) and ‘small town’ are less likely to participate in vaccine trials, as are non-graduates (OR = 0.85) and those of the BAME ethnicity (0.43). Furthermore, groups more likely to participate included males (OR = 1.29), graduates (OR = 1.28) and the 40–49, 50–59 and 60–69 age groups (OR = 1.88, OR = 1.46 and OR = 1.12 respectively). The results also confirm that respondents with no known health issues are more likely to participate in vaccine trials (OR = 1.60). However, young adults (aged 18–39) were less interested in participating in the vaccine trials (OR = 0.57).

In total, 2864 respondents (58.64%) were classified as ‘non-participants’ in vaccine trials, which is defined as those who were unsure or would choose not to participate in trials. Three hundred forty-six respondents (27.60%) would choose not to participate in trials and 1518 (31.10%) respondents were unsure. In the ‘non-participant’ group, females accounted for 73.25% (*n* = 2098), whilst 39% (*n* = 1167) reported diagnosed health issues. 7.75% (*n* = 222) were smokers. Amongst the qualification classifications, university graduates accounted for 36.35% (*n* = 1041), followed by non-graduates (32.09%, *n* = 919). Respondents without formal qualifications only constituted 2.55% (*n* = 73). Concerning the age groups, the number of ‘non-participant’ respondents was highest in the 40–49 (21.41%) and 50–59 (21.72%) age groups. Only 11.33% (*n* = 315) of respondents in the ‘non-uptake’ group were in the age group 70 years and above. The BAME community accounted for 7.23% (*n* = 158) whilst 86.45% (*n* = 818) belonged to the non-BAME community. Of those who would choose not to participate in vaccine trials, 15.22% (*n* = 436) indicated that they have previously declined vaccinations. The details are reported in Tables 4 and 5. Tables 6 and 7 provide the details of the postcode analysis of the non-participant group. ‘Core city’ and ‘small towns’ accounted for the majority of these ‘non-participants’.

**Table 4:** Non-participants in vaccine trials

Vaccine trial			
	Not sure (1518)	Not interested (1346)	Total (2864)
**Gender (%)**			
Male	24.51	26.60	25.49
Female	74.70	71.62	73.25
Prefer not to say	0.79	1.78	1.26
**Smoker (%)**	8.10	7.36	7.75
**Diagnosed health condition (%)**	41.70	36.03	39.04
**Qualification (%)**			
No qualification	3.10	1.93	2.55
Non-graduates	33.99	29.94	32.09
Graduates	35.57	37.22	36.35
Post-graduates	18.64	20.51	19.52
Prefer not to say	8.70	10.40	9.50
**Age group (%)**			
Under 18	0.07	0.15	0.11
18–29	10.68	14.13	12.30
30–39	13.46	18.37	15.77
40–49	21.02	21.84	21.41
50–59	21.88	21.54	21.72
60–69	20.42	13.91	17.36
70 +	12.47	10.05	11.33
**Ethnicity (%)**			
BAME	7.18	7.28	7.23
Non-BAME	89.00	83.58	86.45
Prefer not to say	3.82	9.14	6.32

**Table 5:** Examining the perception of vaccine and its effect on participation in vaccine trials on ethnicity

Questions	Overall		BAME		Non-BAME		BAME vis-à-vis non-BAME	Latent variable	Odds ratio	Std. error (***p-value***)
	Mean score	Std. Dev.	Mean score	Std. Dev.	Mean score	Std. Dev.	(test statistic) ***p***-value			
Vaccines are safe	3.97	0.938	4.12	0.846	3.96	0.904	(1.784) 0.074*	Perception of generic vaccine on overall health	1.67	0.051 *** (0.000)
Vaccines keep you healthy	4.07	0.951	4.19	0.931	4.04	0.916	(1.379) 0.168			
Vaccines are imp for overall health	4.14	0.963	4.27	0.888	4.14	0.965	(1.43) 0.146			
Approved COVID-19 vaccines are safe	4.04	1.019	4.30	0.847	4.03	1.023	(2.803) 0.005***	Perception of COVID-19 vaccine on overall health	1.62	0.044*** (0.000)
Vaccine is a necessity for COVID-19	4.08	1.069	4.24	0.920	4.07	1.035	(1.673) 0.094*			
Vaccine is best to prevent COVID-19	4.17	1.058	4.32	0.840	4.16	1.072	(1.983) 0.050**			
Only vaccine can control COVID-19	4.11	1.098	4.27	0.926	4.10	1.062	(1.890) 0.061*			
COVID-19 vaccine will not harm me	3.94	1.022	4.16	0.854	3.93	1.026	(2.866) 0.005***			
Importance of people involved in vaccine trial	4.31	0.961	4.47	0.785	4.31	0.922	(2.063) 0.041**	Perception of COVID-19 vaccine trials	1.38	0.044*** (0.000)
People from all backgrounds are important to participate in vaccine trials	4.48	0.982	4.59	0.735	4.58	0.868	(1.530) 0.129			

Note: ***Significance is 1% level, **significance at 5% level and *significance at 10% level

**Table 6:** Not interested in trials (postcode breakup)

Not interested in trials	Postcode classifications						Total respondents	Percentage
	Core city	Other city	Large town	Medium town	Small town	Village		
**Gender (%)**								
Male	56	37	3	37	78	147	358	26.60
Female	193	102	6	112	271	280	964	71.62
Prefer not to say	3	5	0	4	9	3	24	1.78
**Smoker (%)**	23	12	0	9	24	31	99	7.36
**Diagnosed health condition (%)**	71	66	3	51	115	179	485	36.03
**Qualification (%)**								
No qualification	3	2	0	1	7	13	26	1.93
Non-university goers	63	38	2	25	117	158	403	29.94
Graduates	93	57	1	77	120	153	501	37.22
Post-graduates	68	28	3	37	74	66	276	20.51
Prefer not to say	25	19	3	13	40	40	140	10.40
**Age group (%)**								
Under 18	0	1	0	1	0	0	2	0.15
18–29	38	14	1	34	58	42	187	13.89
30–39	71	24	2	32	58	56	243	18.05
40–49	70	30	2	41	66	80	289	21.47
50–59	53	29	1	23	88	91	285	21.17
60–69	13	23	2	11	39	96	184	13.67
70 +	5	16	1	7	41	63	133	9.88
Prefer not to say	2	7	0	4	8	2	23	1.71
**Ethnicity (%)**								
AME	78	18	4	33	38	19	190	14.12
Non-BAME	167	122	4	114	314	404	1125	83.58
Prefer not to say	7	4	1	6	6	7	31	2.30

**Table 7:** Unsure about trials (postcode breakup)

Unsure (trial participation)	Postcode classifications				Total respondents	Percentage
	Other city	Medium town	Small town	Village		
**Gender (%)**						
Male	193	3	121	55	372	24.51
Female	383	31	571	149	1134	74.70
Prefer not to say	2	0	9	1	12	0.79
**Smoker (%)**	56	3	49	15	123	8.10
**Diagnosed health condition (%)**	244	13	263	113	633	41.70
**Qualification (%)**						
No qualification	26	0	15	6	47	3.10
Non-university goers	234	13	184	85	516	33.99
Graduates	183	15	279	63	540	35.57
Post-graduates	80	2	172	29	283	18.64
Prefer not to say	55	4	51	22	132	8.70
**Age group (%)**						
Under 18	0	0	1	0	1	0.07
18–29	51	3	97	10	161	10.61
30–39	47	9	127	20	203	13.37
40–49	86	11	181	39	317	20.88
50–59	128	8	144	50	330	21.74
60–69	154	2	96	56	308	20.29
70 +	111	1	47	29	188	12.38
Prefer not to say	1	0	8	1	10	0.66
**Ethnicity (%)**						
BAME	23	3	118	11	155	10.21
Non-BAME	553	31	575	192	1351	89.00
Prefer not to say	2	0	8	2	12	0.79

Table 5 reports the mean scores of respondents’ perceptions of vaccines and their effect on participation in vaccine trials. For the survey, a 5-point Likert scale was used, i.e. strongly disagree (1), disagree (2), neutral (3), agree (4) and strongly agree (5). The mean scores for all the questions are above three. The scores are significantly higher in all of the questions for those respondents who are interested in vaccine trials. Furthermore, it is evident that the standard deviation was very low for this group as compared to those who were not interested in trials (details are provided in Table 8). This indicates that even though the respondents tend to agree on the importance and necessity of COVID-19 vaccine trials, the responses varied considerably. There was a significant difference in the mean scores of the responses between the BAME and the non-BAME community. Mean scores of the former were considerably higher than those of the latter community, although standard deviations were lower. The scores were also significantly different across the postcode classifications. In particular, the mean scores of ‘Medium Town’ were significantly higher than the others for the questions related to vaccine trials. The maximum number of male respondents who were interested in trials belonged in this classification (details are provided in Table 9). Furthermore, the findings show that the odds ratio of ‘perception of COVID-19 vaccine trials’ (OR = 1.38) plays a critical role amongst the respondents along with the perception of generic vaccines and the COVID-19 vaccine in particular.

**Table 8:** Examining the perception of vaccine and its effect on participation in vaccine trials

Questions	Interested		Non-participant		Interested vis-à-vis non-participant
	Mean score	Std. Dev.	Mean score	Std. Dev.	***p***-value
Vaccines are safe	4.260	0.804	3.500	1.097	0.000***
Vaccines keep you healthy	4.323	0.833	3.632	1.121	0.000***
Vaccines are imp for overall health	4.415	0.791	3.648	1.171	0.001***
Approved COVID-19 vaccines are safe	4.400	0.789	3.393	1.214	0.003***
Vaccine is a necessity for COVID-19	4.379	0.868	3.503	1.276	0.000***
Vaccine is best to prevent COVID-19	4.496	0.795	3.522	1.326	0.000***
Only vaccine can control COVID-19	4.428	0.852	3.481	1.349	0.001***
COVID-19 vaccine will not harm me	4.272	0.843	3.356	1.192	0.000***
Importance of people involved in vaccine trial	4.598	0.737	3.873	1.126	0.003***
People from all backgrounds are important to participate in vaccine trials	4.692	0.698	4.126	1.091	0.001***

Note: ***states significance at 1% level

**Table 9:** Examining the perception of vaccine trials vis-à-vis postcode classification (medium town and the rest)

Postcode classification	Importance of people involved in vaccine trial			People from all backgrounds are important		
	Mean difference	Std. error	***p***-value	Mean difference	Std. error	***p***-value
**Core city**	0.238*	0.051	0.067	0.134***	0.048	0.005
**Other city**	0.037***	0.041	0.001	0.016***	0.038	0.000
**Large town**	0.037*	0.080	0.074	0.041*	0.075	0.085
**Small town**	0.215***	0.043	0.000	0.115**	0.041	0.035
**Village**	0.260***	0.045	0.000	0.186**	0.042	0.045

Note: ***significant at 1% level and *10% significance level

## Discussion

This study is the largest and first population-based study in the UK regarding COVID-19 vaccination and vaccination trial perceptions. Our results show that less than half of the respondents (41.4%) are interested in partaking in vaccination trials. Interestingly, the UK COVID-19 vaccine registry shows that only 357,706 participants have registered as of the 1st December, which is estimated to be only 0.5% of the UK population [13, 14]. Furthermore, only 6 out of 1518 UK COVID-19 studies were collecting data on ethnicity.

Interestingly, this is the first study to identify that ‘other cities’, smaller cities, such as Leicester and Aberdeen, are more likely to participate in trials compared to larger metropolitan ‘core cities’, such as London and Birmingham. A reason for this could be attributed to ‘core cities’ having greater pockets of inner-city poverty and health inequalities, compared to ‘outer cities’ [15].

The BAME community are less likely to get involved in the COVID-19 vaccination trials, despite them being at higher risk of COVID-19. This correlates with our study results. The UK COVID-19 vaccine registry also demonstrates that BAME groups are short of reaching national targets representing less than 8% of the registry despite representing 13% of the population [13]. Of note, the Black population only consisted of 0.5% of the total number. Trials have historically struggled with gaining a diverse population with greater intervention required to engage ethnic minority groups into trials [16]. In US studies, the Black population have been significantly under-represented in clinical trials [17]. The under-representation, of the Black community in particular, is likely to be attributed to mistrust in the medical profession, as well as historical oppression and health inequalities [18, 19]. A recent study found that only 14% of Black adults trust that a vaccine would be safe and fewer than half of the Black adults would accept a licenced COVID-19 vaccine even if freely available [20].

Our findings suggest that mistrust is a key factor in non-uptake for vaccination trials. Free text comments from the survey revolved around the idea of the BAME community being used as ‘guinea pigs’ for trials to verify vaccine results, and mistrust around government strategies. Similar sentiments have been found in US studies [17, 20, 21]. This was likely to have taken influence from social media views at the time of survey completion, particularly those highlighting adverse events from vaccine trials and vaccine trials being rushed. Furthermore, there are greater anti-vaccination sentiments shared on social media and are spread quicker compared to positive ones promoting trial uptake [22]. Multiple studies have highlighted a number of other reasons as to why trials tend to disadvantage minorities from attending. This includes poor health literacy, hidden costs related to reaching trials, lack of knowledge about the condition being studied, distrust in the research process and the researchers, and language barriers [8, 23].

Whilst the highest proportion of BAME respondents were in cities, there seems to be even less interest from the BAME community in ‘core cities’ compared to ‘other cities’. There is a greater proportion of the BAME community residing in the inner city groups of large cities and these areas tend to have poorer health outcomes and suffer greater health inequalities [24, 25], whereas BAME groups in smaller cities are, however, more likely to consider partaking in trials as these areas are likely to be less economically deprived and tend to have more educated BAME groups [25].

In our study, the over 70s group was the least willing to partake in vaccination trials. For the vaccine registry, as of November 2020, over 80s consist of only 1% of COVID-19 vaccination trial participants [13]. This is despite providing much needed diversity and clinical benefit compared to younger and healthier participants. Although the increased risk of morbidity and poly-pharmacy brings unique challenges of how effective the vaccine could be, participation from the elderly can render the trials more generalisable. Unfortunately, elderly patients tend to have greater refusal rates than the younger population and many do not actively seek out clinical trials or are even informed of the availability of clinical trials [8].

There are multiple reasons as to why the elderly hesitate to participate in clinical trials. Many within the elderly population do not understand the possible benefits of the research being undertaken. Informed consent in the elderly can also be complicated by the possibility of cognitive impairment. Transportation difficulties are consistently cited as a primary concern and a barrier for elderly adults considering participation in a research study whilst mobility issues could also potentially make follow-up visits difficult [8].

In our study, the majority of those over 60s who were not interested in participating in trials were from ‘small towns’ and ‘villages’, which are known as more rural areas. Participation in clinical trials in rural areas is significantly lower [26, 27]. Often, these areas have a greater older population and are further away from trial sites, making it harder to access. Furthermore, rural participants are likely to be less aware about vaccine trials and have more misperceptions than inner city participants [21].

The perception of the COVID-19 vaccine can play a key role in deciding whether an individual will partake in a vaccine trial. Our results showed that the 40–59 age group has the greatest interest in participating in vaccination trials. There is also interest from those who had no health conditions. These younger and healthier adults may be motivated by altruism and may see the societal benefits of vaccine research, surpassing any personal health risks [28, 29]. Our study found that if one was interested in partaking in the vaccination trial, then they are also likely to have a positive perception of the COVID-19 vaccine on overall health and of the vaccine trials. Furthermore, they are more likely to agree on the importance of having a variety of people, of all backgrounds, to participate in vaccine trials.

Despite the majority of our respondents being female, younger males were more interested in partaking in vaccine trials. Historically, women, particularly of child bearing age, are harder to recruit for vaccination trials [30]. Studies have shown that females, particularly those with underlying health issues, can also have more distrust in pharmaceutical companies and have been previously under-represented in other respiratory trials [10].

### Future challenges

The challenges with recruitment for trials are set to increase, as phase 3 vaccine trials continue to take place from 2021 [31]. Barriers will become more pronounced when recruiting to placebo phase 3 trials and further non-inferiority studies, where vaccines will go head to head. Thus, there is a need to consider the redesign and reshaping of these studies to consider these barriers, and engage the patients in the recruitment plan for these newly designed studies.

Vulnerable groups, such as the BAME and elderly, are most likely to receive an approved vaccine first so would be hesitant to partake in non-approved vaccine trials. It may also be considered unethical to have these high-risk groups involved in testing once there is already an approved vaccine. In terms of the young population, they may well be one of the last groups to receive an approved vaccine, and so may be more drawn towards involvement in trials.

The UK will also be the first country to do human challenge trials with COVID-19 in 2021 [32]. The challenge trial will involve infecting healthy participants with the COVID-19 virus in a controlled environment and then being administered the vaccine [33]. Eligible participants must not have any previous health conditions so that they experience only a mild infection [29, 32]. Consequently, high-risk groups such as the elderly are likely to be excluded from these trials [29].

Whilst our study shows willingness from those who have no health conditions, more work still needs to be done in recruiting high numbers of healthy young participants to adequately power these challenge trials. In our study, the odds ratio for those under 40 was less than 1, suggesting that there is a large proportion of young people who are disinterested in participating in trials. This is somewhat surprising, as previous studies showed a high willingness (64%) towards vaccination trials, particularly in university students [28]. More worrying, any adverse events with these challenge trials are vulnerable to negatively tipping the balance in vaccine uptake rates.

### Tackling barriers towards trial recruitment

This study has shown that there is a clear need for launching national awareness and education campaigns. The aim would be to improve the public’s knowledge about the burden of diseases and the need for vaccine development, thereby harnessing the public’s motivation to take part [11]. Moreover, campaigns will need to tackle issues of mistrust whilst remaining cautious with economic coercion that would aim to drive participation in the economically marginalised [28].

There may be a need to bring the trial to the subject where distance is a barrier. Mobile units could be formed to conduct study visits remotely. This would eliminate the need for the subject to find transport to the clinical trial site and reduce travel time, therefore reducing the impact on the subject’s daily commitment [11]. Alternatively, the use of telemedicine approaches has become progressively more popular and acceptable by health authorities, medical doctors and patients after the current pandemic [34].

Researchers should remain flexible in their approach and incorporate different types of media and community resources to enhance recruitment. Community engagement techniques are being increasingly used by the NIHR, such as the INCLUDE initiative. This initiative has been formed to ensure there is adequate representation of under-served groups, which will be done through careful funding and regulatory approval [35]. Informed consent for participation in a clinical trial should be kept simple and short to ensure adequate understanding of the subject, yet comprehensive to ensure useful information can be collected whilst preserving ethical principles of informed consent [8]. Consent should be made possible to be done in various languages. Other solutions can involve having more minority researchers conducting the trials. Having greater diversity in principal investigators can be beneficial with this. This may curtail bias in recruitment of participants from under-represented populations and allow for improved communication during recruitment [17, 20, 23].

It is important to engage General Practitioners (GP) into vaccine trial recruitment, where they can act as key facilitators for older patients and BAME involvement in trials [8, 20, 21]. Not only can GPs offer a more personalised approach, but they can also facilitate in building greater awareness, as often the barrier is a lack of awareness of trials [8]. However, a barrier to this can be a lack of confidence in GPs being able to recruit trial participants. This can be overcome by establishing formal training for GPs on discussing clinical trials with patients and with specific patient populations to facilitate improved shared decision-making [17].

## Limitations

A limitation of this study is that we were not able to ascertain the reasons for those not wanting to partake in vaccination trials. We were only able to deduce their general perception towards COVID-19 and vaccines, as well as extracting demographic and geographical data. Being able to understand the key reasons would be beneficial in targeting educational campaigns to tackle specific barriers to trial recruitment.

This is one of the most BAME inclusive COVID-19 vaccination-related studies in the UK. However, our BAME participant percentage (9.44%) is still below the overall BAME proportion in the UK, which is approximately 13% [35]. In particular, we received a very small amount of Black and East Asian (e.g. Chinese) participants, so this makes it difficult to deduce the views of these communities. This reflects the need for further work to engage the Black and East Asian ethnic groups into research in general.

Similar to other published surveys, as this was an online survey completed via a computer or smartphone, there was also selection bias. This would exclude those with a lack of digital literacy, which could include the older population and economically marginalised groups, who are already known to not engage with the UK National Health Services digital resources [18]. This survey is more likely to attract responses from those who have stronger opinions related to the COVID-19 vaccination and may be more self motivated to complete this survey.

## Conclusion

Our study shows a trial uptake demography that proves a challenge for future phase 3 trials. The vaccine trials should represent a diverse participant population in terms of age, ethnicity and comorbidities. Missing these targets can lead to reduced validity of the study results and can often slow down drug development leading to costly delays. Currently, the UK registry has a very low trial participant uptake on the elderly and BAME population — two high-risk priority groups. It is alarming that these high-risk groups may be losing out on the opportunity to gain so much from this research, including the opportunity to receive lifesaving treatment.

There is a need to design interventional and public health strategies to engage and encourage trial participation from specific demographic groups, such as the BAME community and those aged over 70 population. Using data from the Office of National Statistics can help provide a tailored approach [36]. Our data provides unique insights into participation interest geographically and can be used to target ongoing and future campaigns in rural and core inner city populations. Our study provides possible interventions to increase the uptake for COVID-19 vaccine trial participations with the overall goal to acquire a safe and effective vaccine. This can provide useful in future trials that will continue on for 2021, such as human challenge trials, phase 3 trials and non-inferiority COVID-19 vaccine studies.

## Supplementary Information

**Additional file 1.** Screenshots of Survey.

## Data Availability

Cross-sectional data can be made available (de-identified participant data) after authors’ review of the request.

## References

[1] Li Q, Guan X, Wu P, Wang X, Zhou L, Tong Y, Ren R, Leung KSM, Lau EHY, Wong JY, Xing X, Xiang N, Wu Y, Li C, Chen Q, Li D, Liu T, Zhao J, Liu M, Tu W, Chen C, Jin L, Yang R, Wang Q, Zhou S, Wang R, Liu H, Luo Y, Liu Y, Shao G, Li H, Tao Z, Yang Y, Deng Z, Liu B, Ma Z, Zhang Y, Shi G, Lam TTY, Wu JT, Gao GF, Cowling BJ, Yang B, Leung GM, Feng Z (2020). Early transmission dynamics in Wuhan, China, of novel coronavirus-infected pneumonia. N Engl J Med.

[2] Coronavirus COVID-19 dashboard by the center for systems science and engineering (CSSE). USA: John Hopkins University. 2020. https://coronavirus.jhu.edu/map.html. Accessed 1 Apr 2021.

[3] Huang C, Wang Y, Xingwang L (2020). Clinical features of patients infected with 2019 novel coronavirus in Wuhan, China. Lancet.

[4] Rothan HA, Byrareddy SN (2020). The epidemiology and pathogenesis of coronavirus disease (COVID-19) outbreak. J Autoimmun.

[5] Gandhi M, Yokoe DS, Havlir DV (2020). Asymptomatic transmission, the Achilles’ heel of current strategies to control Covid-19. N Engl J Med.

[6] Alwan NA, Burgess RA, Ashworth S, Beale R, Bhadelia N, Bogaert D, Dowd J, Eckerle I, Goldman LR, Greenhalgh T, Gurdasani D, Hamdy A, Hanage WP, Hodcroft EB, Hyde Z, Kellam P, Kelly-Irving M, Krammer F, Lipsitch M, McNally A, McKee M, Nouri A, Pimenta D, Priesemann V, Rutter H, Silver J, Sridhar D, Swanton C, Walensky RP, Yamey G, Ziauddeen H (2020). Scientific consensus on the COVID-19 pandemic: we need to act now. Lancet.

[7] Wang J, Jing R, Lai X, Zhang H, Lyu Y, Knoll MD, Fang H (2020). Acceptance of COVID-19 vaccination during the COVID-19 pandemic in China. Vaccine.

[8] Ridda I, MacIntyre CR, Lindley RI (2010). Difficulties in recruiting older people in clinical trials: an examination of barriers and solutions. Vaccine.

[9] Swan J, Robertson M & Evans S. Managing clinical research in the UK: summary of findings. Warwick Business School; 2009. https://qmro.qmul.ac.uk/xmlui/bitstream/handle/123456789/1014/Robertson%20managing%20clinical%20research%202010%20Published.pdf?sequence=2&isAllowed=y. Accessed 26 Nov 2020.

[10] Pahus L, Suehs CM, Halimi L, Bourdin A, Chanez P, Jaffuel D, Marciano J, Gamez AS, Vachier I, Molinari N (2020). Patient distrust in pharmaceutical companies: an explanation for women under-representation in respiratory clinical trials?. BMC Med Ethics.

[11] Harrington L, Van Damme P, Vandermeulen C (2017). Recruitment barriers for prophylactic vaccine trials: a study in Belgium. Vaccine.

[12] City & town classification of constituencies & local authorities. UK Parliament House of Commons Library; 2020 https://commonslibrary.parliament.uk/research-briefings/cbp-8322/. Accessed 26 Nov 2020.

[13] Coronavirus vaccine studies volunteers dashboard. NHS Digital. 2020. https://digital.nhs.uk/dashboards/coronavirus-covid-19-vaccine-studies-volunteers-dashboard-uk. Accessed 26 Nov 2020.

[14] Population estimates. Office for National Statistics. 2020. https://www.ons.gov.uk/peoplepopulationandcommunity/populationandmigration/populationestimates. Accessed 26 Nov 2020.

[15] Naylor C. London and health: the best and worst of cities. The Kings Fund; 2017. https://www.kingsfund.org.uk/blog/2017/08/london-and-health-best-and-worst-cities. Accessed 26 Nov 2020.

[16] McGarry ME, McColley SA (2016). Minorities are underrepresented in clinical trials of pharmaceutical agents for cystic fibrosis. Ann Am Thorac Soc.

[17] Hamel LM, Penner LA, Albrecht TL, Heath E, Gwede CK, Eggly S (2016). Barriers to clinical trial enrollment in racial and ethnic minority patients with cancer. Cancer Control.

[18] Yancy CW (2020). COVID-19 and African Americans. JAMA.

[19] Jaklevic MC (2020). Researchers strive to recruit hard-hit minorities into COVID-19 vaccine trials. JAMA.

[20] Langer Research Associates (2020). COVID collaborative survey: coronavirus vaccination hesitancy in the Black and Latinx communities.

[21] Geana M, Erba J, Krebill H (2016). Searching for cures: inner-city and rural patients’ awareness and perceptions of cancer clinical trials. Contemp Clin Trials Commun.

[22] Puri N, Coomes EA, Haghbayan H, Gunaratne K (2020). Social media and vaccine hesitancy: new updates for the era of COVID-19 and globalized infectious diseases. Hum Vaccin Immunother.

[23] Chastain DB, Osae SP, Henao-Martínez AF, Franco-Paredes C, Chastain JS, Young HN (2020). Racial disproportionality in COVID clinical trials. N Engl J Med.

[24] Finney N & Lymperopoulou K. Local ethnic inequalities: ethnic differences in education, employment, health and housing in districts of England and Wales, 2001–2011. Runnymede Report. The University of Manchester in Association with the Runnymede Trust. https://www.runnymedetrust.org/uploads/Inequalities%20report-final%20v2.pdf. Accessed 26 Nov 2020.

[25] Garner S, Bhattacharya G (2011). Poverty, ethnicity and place. JRF programme paper: poverty and ethnicity.

[26] Friedman DB, Foster C, Bergeron CD, Tanner A, Kim SH (2015). A qualitative study of recruitment barriers, motivators, and community-based strategies for increasing clinical trials participation among rural and urban populations. Am J Health Promot.

[27] Baquet CR, Commiskey P, Daniel Mullins C, Mishra SI (2006). Recruitment and participation in clinical trials: socio-demographic, rural/urban, and health care access predictors. Cancer Detect Prev.

[28] Sun S, Lin D, Operario D (2020). Interest in COVID-19 vaccine trials participation among young adults in China: willingness, reasons for hesitancy, and demographic and psychosocial determinants.

[29] McPartlin SO, Morrison J, Rohrig A (2020). Covid-19 vaccines: should we allow human challenge studies to infect healthy volunteers with SARS-CoV-2?. BMJ.

[30] Liu KA, Mager NA (2016). Women’s involvement in clinical trials: historical perspective and future implications. Pharm Pract.

[31] Janssen to begin COVID-19 vaccine trials in the UK. UK Government Department for Business, Energy & Industrial Strategy. 2020. https://www.gov.uk/government/news/janssen-to-begin-covid-19-vaccine-trials-in-the-uk. Accessed 28 Nov 2020.

[32] Kirby T (2020). COVID-19 human challenge studies in the UK. Lancet Respir Med.

[33] Sheets R & Knezevic I. Human challenge trials for vaccine development: regulatory considerations. Expert committee on biological standardization. World Health Organisation; 2016. https://www.who.int/biologicals/expert_committee/Human_challenge_Trials_IK_final.pdf. Accessed 26 Nov 2020.

[34] Waterhouse DM, Harvey RD, Hurley P, Levit LA, Kim ES, Klepin HD, Mileham KF, Nowakowski G, Schenkel C, Davis C, Bruinooge SS, Schilsky RL (2020). Early impact of COVID-19 on the conduct of oncology clinical trials and long-term opportunities for transformation: findings from an American Society of Clinical Oncology survey. JCO Oncol Pract.

[35] Anderson E, Treweek S, Oshisanya A, Witham M. Serving the under-served – how NIHR’s INCLUDE initiative will make trials better reflect all members of society. National Institute for Health Research; 2020. https://www.nihr.ac.uk/blog/serving-the-under-served-how-nihrs-include-initiative-will-make-trials-better-reflect-all-members-of-society/26289. Accessed 30 Nov 2020.

[36] Office for National Statistics. Population of England and Wales. 2020. https://www.ethnicity-facts-figures.service.gov.uk/uk-population-by-ethnicity/national-and-regional-populations/population-of-england-and-wales/latest. Accessed 26 Nov 2020.

