# Supplementary material for: The UPTAKE study: implications for the future of COVID-19 vaccination trial recruitment in UK and beyond
Source: Trials. 2021 Apr 20;22:296. doi: 10.1186/s13063-021-05250-4 (PMC8057665; doi:10.1186/s13063-021-05250-4)

**Additional file 1: Screenshots of Survey**

This form was completed via Google Forms, and at different stages of completing the form, one was redirected to the next page with a progress bar underneath. Below are the screenshots.


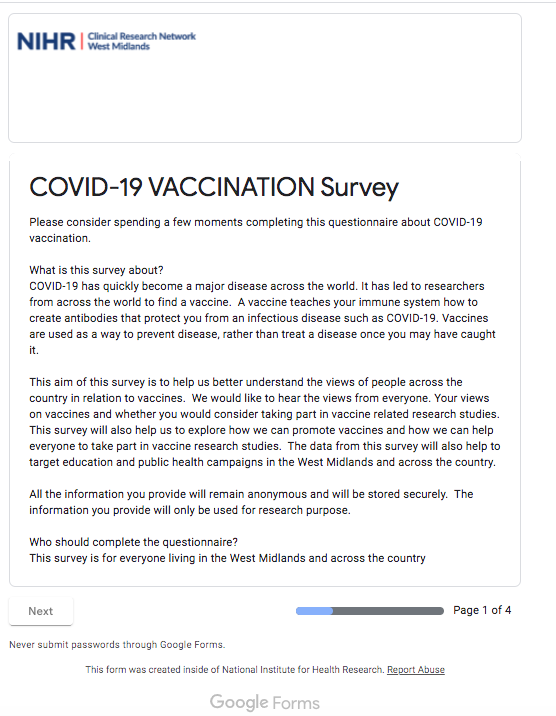


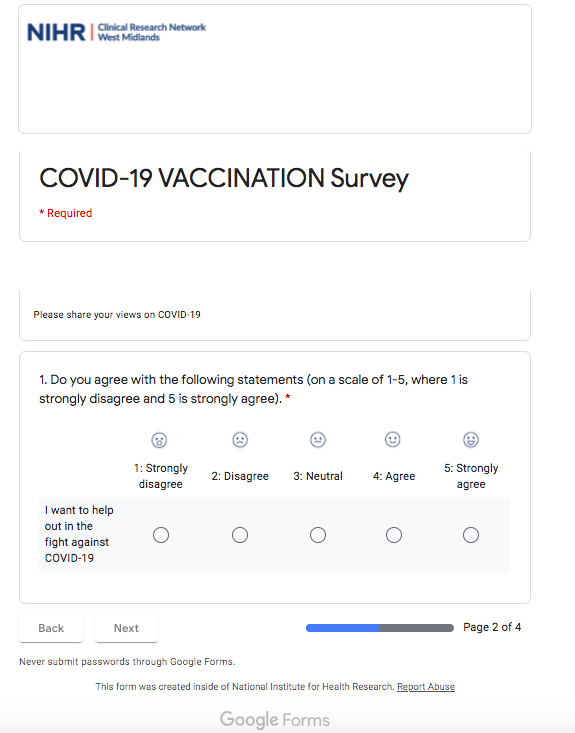


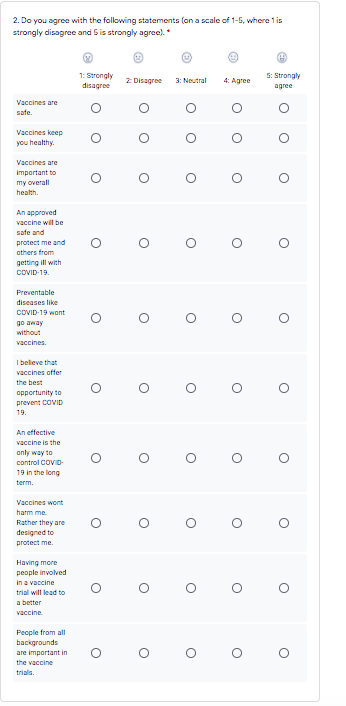


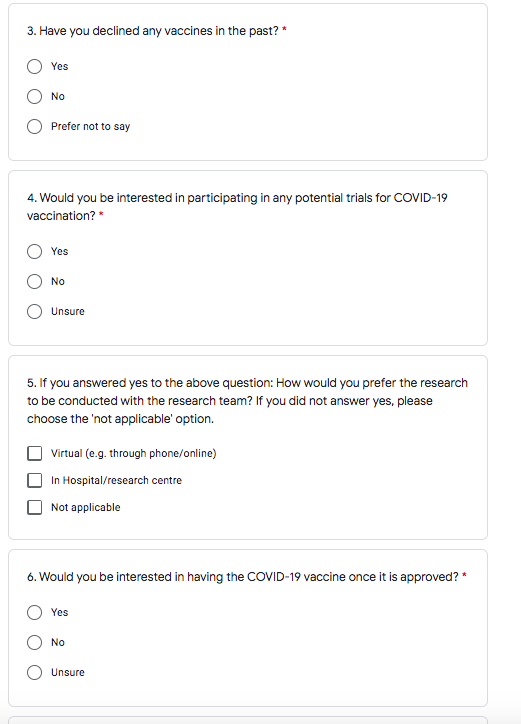


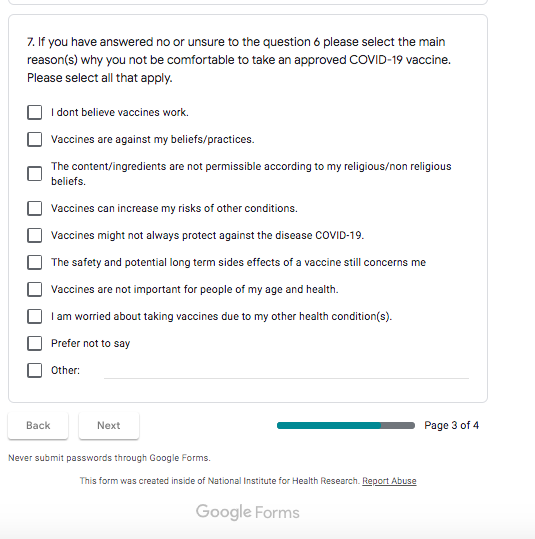


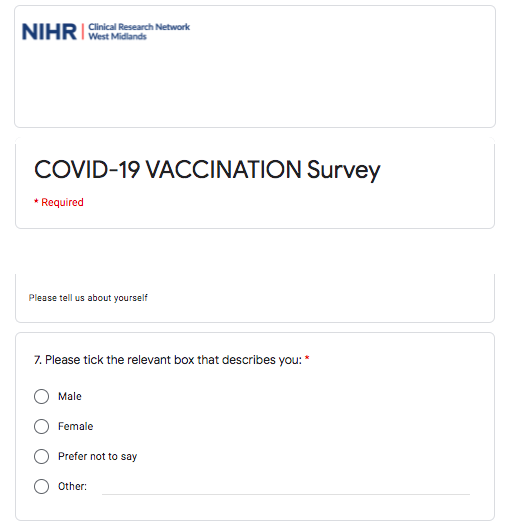


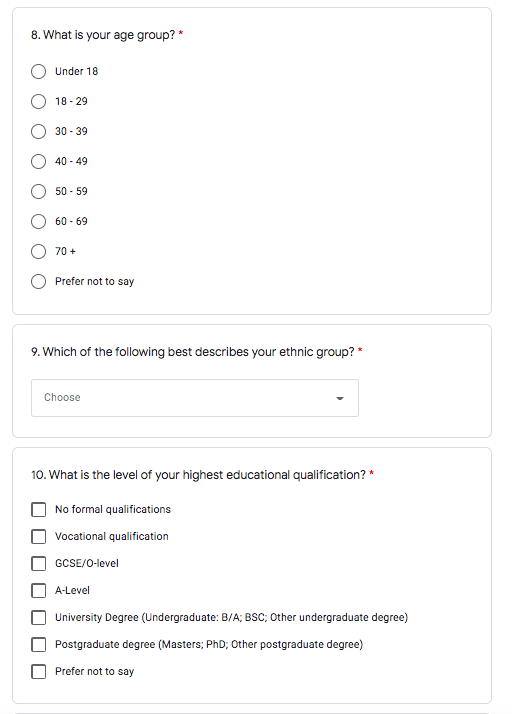


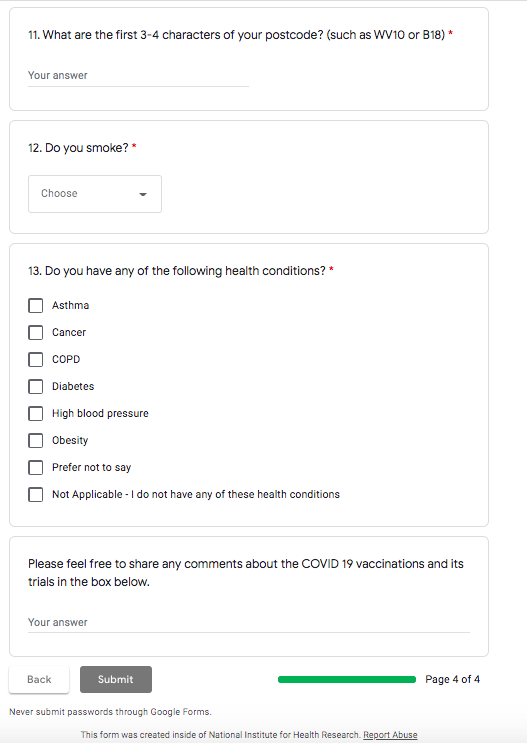


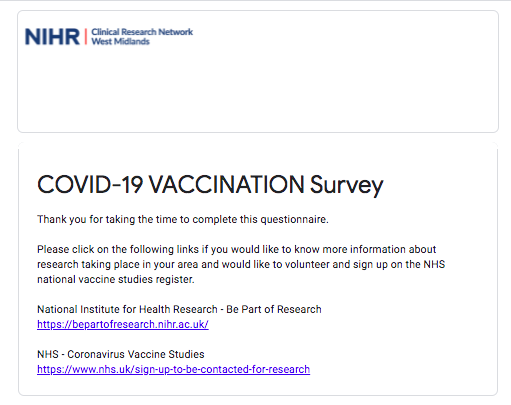

Supplement: Supplementary file 1 — Additional file 1. Screenshots of Survey. [file 13063_2021_5250_MOESM1_ESM.docx]
